# Comparative Safety Profiles of Biosimilars vs. Originators Used in Rheumatology: A Pharmacovigilance Analysis of the EudraVigilance Database

**DOI:** 10.3390/jcm14051644

**Published:** 2025-02-28

**Authors:** Victoria Nikitina, Greta Santi Laurini, Nicola Montanaro, Domenico Motola

**Affiliations:** 1 Unit of Pharmacology, Department of Medical and Surgical Sciences, Alma Mater Studiorum University of Bologna, Via Irnerio 48, 40126 Bologna, Italy; victoria.nikitina2@unibo.it (V.N.);; 2 Alma Mater Studiorum University di Bologna, 40126 Bologna, Italy

**Keywords:** biosimilars, rheumatology, adalimumab, infliximab, rituximab, etanercept, safety, pharmacovigilance, post-marketing

## Abstract

**Background**: The advent of biosimilars has revolutionized the management of conditions like rheumatoid arthritis by offering cost-effective alternatives to expensive biologics. **Objectives**: This study aims to compare the post-marketing safety profiles of biosimilars used in rheumatology with their respective reference products (RPs). **Methods**: Data were retrieved from EudraVigilance for biosimilars of adalimumab, etanercept, infliximab, and rituximab, and compared with their RPs. Our analysis focused on biosimilars authorized before 2021, using data from January 2021 to December 2023. We conducted a descriptive analysis of suspected adverse events, categorized using the Medical Dictionary for Regulatory Activities, and performed a comparative analysis using the reporting odds ratio to identify potential safety signals of disproportionate reporting. **Results**: We analyzed 75,327 reports, identifying 566,249 drug–event pairs. The results indicate that biosimilars have safety profiles largely comparable to their RPs. Female patients predominated in the reports, representing 69.4% of RPs and 56.9% of biosimilars. Notably, biosimilars demonstrated higher reporting rates for non-serious suspected adverse drug events (AEs), such as injection site pain, arthralgia, and fatigue. Specific AEs, including drug ineffectiveness and off-label use, were more frequent for infliximab and etanercept biosimilars, possibly reflecting real-world usage patterns and nocebo effects. Serious AEs, including malignancies and immunological reactions, were also noted, underscoring the necessity for ongoing monitoring. **Conclusions**: Our findings suggest that biosimilars are safe alternatives to RPs, contributing to significant healthcare cost savings in the EU. This study underscores the need for ongoing pharmacovigilance and long-term safety research to validate the clinical use of biosimilars in rheumatology.

## 1. Introduction

Disorders like rheumatoid arthritis, psoriatic arthritis, ankylosing spondylitis, and others have posed significant challenges in terms of effective management. However, for nearly 30 years, the introduction of biological medications, also known as biologics, has significantly improved outcomes for numerous patients across various fields, including those with rheumatic diseases [1]. Given the complex structure of biologics and their production in living systems under rigorously monitored conditions, their development and manufacturing entail substantial costs, which are often inaccessible for healthcare systems [2,3]. After their exclusive patents expired, numerous biopharmaceutical companies have introduced to the market similar biologic products, known as biosimilars. This has led to a considerable reduction in prices, making these treatments more cost effective. The European Medicines Agency (EMA) defines a biosimilar as “a biological medicine highly similar to another already approved biological medicine (the ‘reference medicine’). Biosimilars are approved according to the same standards of pharmaceutical quality, safety and efficacy that apply to all biological medicines” [4]. Other major international regulatory agencies have also provided definitions of biosimilars, as detailed in Table 1.

The EMA further emphasizes that biosimilars are highly similar to the reference product in terms of structure, biological activity, efficacy, safety, and immunogenicity profile [4]. Like other regulatory agencies, the EMA has established specific comparability pathways to confirm the similarity between a biosimilar candidate and its reference product (RP) [7,8,9]. Biosimilars differ from generics of chemical products because it is not possible to develop molecules identical to their reference products due to natural biological variation and heterogeneous production processes [10]. Hence, it is essential that biosimilars are comparable to their reference biologics in terms of safety.

Adalimumab, etanercept, infliximab, and rituximab are key biologic drugs used in the treatment of various rheumatological conditions. Adalimumab, infliximab, and rituximab are monoclonal antibodies (mAbs): adalimumab is employed in the treatment of several conditions, including rheumatoid arthritis, juvenile idiopathic arthritis, axial spondyloarthritis, and psoriatic arthritis [11]; infliximab is primarily used for treating rheumatoid arthritis, ankylosing spondylitis, and psoriatic arthritis [12]; and rituximab is indicated for the treatment of rheumatoid arthritis and certain autoimmune conditions, such as granulomatosis with polyangiitis and microscopic polyangiitis [13]. Etanercept, a human tumor necrosis factor receptor p75 Fc fusion protein, is utilized in the treatment of rheumatoid arthritis, juvenile idiopathic arthritis, psoriatic arthritis, and axial spondylarthritis [14].

An increasing array of biosimilar variants for these agents is accessible on the market, with numerous others undergoing development. Just like with any medication, it is crucial to conduct post-marketing surveillance to assess the safety profiles of both biologics and biosimilars. The purpose of this study was to examine the post-marketing pharmacovigilance data of biosimilars used in rheumatic diseases and compare them to the respective RPs.

## 2. Materials and Methods

The data were extracted from the EudraVigilance (EV) database, via adrreports.eu (http://www.adrreports.eu/, accessed on 1 January 2024). EV is a public spontaneous reporting system, maintained by the EMA on behalf of the European Union (EU), which receives Individual Case Safety Reports (ICSRs) of suspected adverse drug reactions (ADRs) within the European Economic Area (EEA). As the processing of the reports does not include a causality assessment, it is more appropriate to define all suspected ADRs as adverse events (AEs) and will be referred to as such throughout the remainder of this manuscript.

We extracted all the ICSRs related to the biosimilars of 4 biological medicines reported as suspected drugs, namely adalimumab, etanercept, infliximab, and rituximab; then, we compared their safety profiles to the corresponding RPs (*Humira*^®^, *Enbrel*^®^, *Remicade*^®^, and *MabThera*^®^, respectively). The EU-licensed biosimilars authorized before the year 2021 were included: 7 different biosimilar adalimumabs (*Amgevita*^®^, *Amsparity*^®^, *Hulio*^®^, *Hyrimoz*^®^, *Hefiya*^®^, *Idacio*^®^, *Imraldi*^®^), 3 biosimilar etanercepts (*Benepali*^®^, *Erelzi*^®^, *Nepexto*^®^), 4 biosimilar infliximabs (*Flixabi*^®^, *Inflectra*^®^, *Remsima*^®^, *Zessly*^®^), and 5 biosimilar rituximabs (*Blitzima*^®^, *Rixathon*^®^, *Riximyo*^®^, *Ruxience*^®^, *Truxima*^®^). Table 2 lists these medicine products with their respective date of approval and the patent expiry date for the corresponding reference product.

Retrospective analyses were performed on the biosimilars that have spent more time on the market, and taking into account the fact that 3 of them received marketing authorization valid throughout the European Union in 2020 (*Amsparity*^®^ on 13 February 2020, *Nepexto*^®^ on 20 May 2020, and *Ruxience*^®^ on 1 April 2020), we considered the 3 year period between 1 January 2021 and 31 December 2023, aiming to analyze as many biosimilars as possible over the same period of time.

### 2.1. Descriptive Analysis

The extracted reports were identified by a unique EU Local Number. The ICSRs contain information on the report type (spontaneous or from clinical studies), primary source qualification (healthcare professional or non-healthcare professional), EV gateway receipt date, patient sex and age group, MedDRA preferred terms (PTs), seriousness criteria, and suspect and concomitant drugs. All the AEs are categorized using the Medical Dictionary for Regulatory Activities (MedDRA). MedDRA is a specific standardized dictionary of medical terminology, designed to facilitate the sharing of regulatory information internationally for medical products. MedDRA terms are arranged in a 5-tiered multi-axial hierarchy, which provides increasing specificity as one descends the scale. At the top level of the hierarchy, there are 27 System Organ Classes (SOCs), which incorporate at the lower levels, High-Level Group Terms (HLGTs) and High-Level Terms (HLTs). Each member of the next level, namely preferred terms (PTs), is a distinct descriptor (single medical concept) for a symptom, sign, disease diagnosis, therapeutic indication, investigation, surgical or medical procedure, and medical social or family history characteristic. Finally, one or more Lower-Level Terms (LLTs) correspond to each PT; the LLTs are effectively entry terms that include synonyms and lexical variants (https://admin.meddra.org/sites/default/files/guidance/file/intguide_26_0_English.pdf, accessed on 23 January 2024). One or more symptoms can be reported for each EV ICSR. We analyzed all the ICSRs related to the RPs and their biosimilars, which are included in this study, performing a descriptive analysis. For each medicine, the notoriety of the suspected AEs was ascertained by checking whether the most frequently reported AEs were listed in the corresponding Summary of Product Characteristics (SPCs) [11,12,13,14,15,16,17,18,19,20,21,22,23,24,25,26,27,28,29,30,31,32,33].

### 2.2. Statistical Analysis

We performed a comparative analysis using a disproportionality method, based on the calculation of the reporting odds ratio (ROR), with a 95% confidence interval (CI). The ROR is commonly used by drug safety professionals to help identify suspected AEs associated with drugs that are reported more frequently than expected. The ROR enables a quantitative approach to be followed, using 2 × 2 contingency tables, comparing the frequency of a drug–event pair for biosimilars vs. the respective RPs. An increased frequency for the drug–event pair can be assumed if the ROR is >1. Biosimilars and their respective RPs were analyzed separately. We included in our analysis only suspected AEs that were found to be statistically significant. We set up our analysis according to the guidelines on signal detection and management in pharmacovigilance, established by the EMA, as follows: the lower bound of the 95% CI is greater than 1; the number of AEs is greater than or equal to 3 for active substances contained in medicinal products included in the additional monitoring list, in accordance with REG Art 23 (see GVP Module X), unless the sole reason for inclusion in the list is the request for a post-authorization safety study (PASS); the number of AEs is greater than 5 for the other active substances; and the event belongs to the Important Medical Event (IME) list, version 26.1 [34]. The data analyses were performed using Excel (Excel, Microsoft Office 365).

## 3. Results

### 3.1. Descriptive Analysis

**Figure 1:**
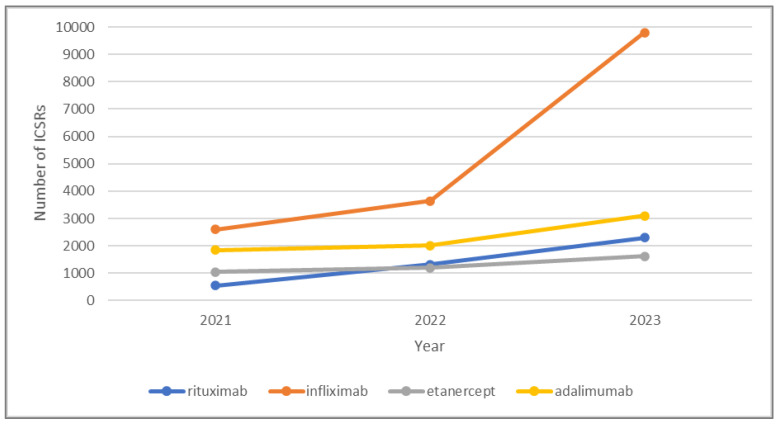
Number of ICSRs for adalimumab, etanercept, infliximab, and rituximab biosimilars in 2021, 2022, and 2023.

**Figure 2:**
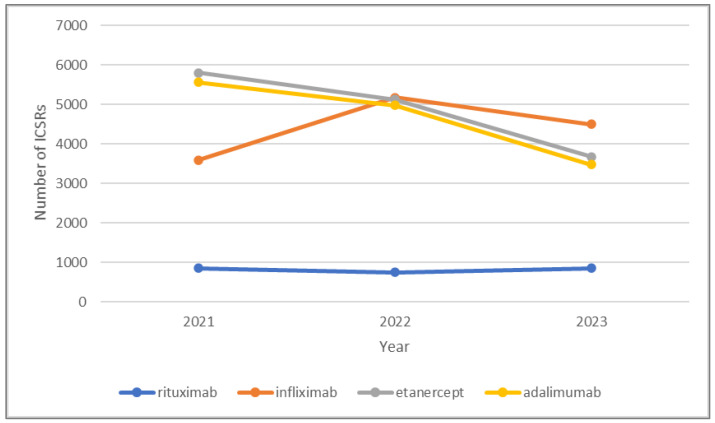
Number of ICSRs for adalimumab (*Humira*^®^), etanercept (*Enbrel*^®^), infliximab (*Remicade*^®^), and rituximab (*MabThera*^®^) originators in 2021, 2022, and 2023.

Figure 1 and Figure 2 show the number of ICSRs for adalimumab, etanercept, infliximab, and rituximab biosimilars and their respective RPs over the study period.

Table 3, Table 4, Table 5 and Table 6 describe the characteristics of each ICSR according to the sex of the patient (female, male, and unknown), age group (0–17 years, 18–64 years, 65–85 years, over 85 years, and unknown), and type of reporter (healthcare professional and non-healthcare professional), for all the medicinal products in this study. A total of 75,327 reports were retrieved for these products: 44,289 reports (58.8%) referred to the original products and 31,038 (41.2%) to the biosimilars.

A total of 566,249 reported PTs were analyzed, 350,721 (61.9%) of which referred to original products, while the remaining 215,528 PTs referred to biosimilars.

The proportion of reports for females was higher than for males for both categories: 69.4% vs. 28.0% for RPs, and 56.9% vs. 37.8% for biosimilars. However, in a small percentage of reports, 2.5% for originators and 5.3% for biosimilars, the information on the sex of the patient was missing. Most of the reports referred to patients aged 18–64 years, followed by those in the age group 65–85 years, while 22.1% of the reports concerning originator biologics and biosimilars were missing information about the patients’ age. Approximately 77% of the reports were submitted by healthcare professionals.

### 3.2. Statistical Analysis

All 75,327 safety ICSRs were examined, generating 566,249 drug–event pairs. We excluded reported AEs referring to incorrect product storage, routine laboratory tests, or incorrect administration, as they were not pertinent to our analysis. Overall, most of the frequently reported and statistically significant AEs for the biosimilars were non-serious and were listed in the corresponding SPCs. Adalimumab biosimilars vs. *Humira*^®^: *drug ineffective n* = 838 events, ROR 1.20 [CI 95% 1.12–1.28]; *injection site pain n* = 450, ROR 9.70 [CI 95% 8.79–10.70]; *arthralgia n* = 440, ROR 1.52 [CI 95% 1.39–1.67]. Etanercept biosimilars vs. *Enbrel*^®^: *fatigue n* = 685 events, ROR 1.17 [CI 95% 1.09–1.26]; *alopecia n* = 615, ROR 1.34 [CI 95% 1.24–1.45]; *arthropathy n* = 551, ROR 1.23 [CI 95% 1.13–1.34]. Infliximab biosimilars vs. *Remicade*^®^: *off-label use n* = 10,028 events, ROR 1.33 [CI 95% 1.31–1.36]; *condition aggravated n* = 7281, ROR 1.78 [CI 95% 1.74–1.83]; *arthralgia n* = 3439, ROR 1.33 [CI 95% 1.28–1.37]. Rituximab biosimilars vs. *MabThera*^®^: *off-label use n* = 2064 events, ROR 1.75 [CI 95% 1.67–1.84]; *condition aggravated n* = 340, ROR 13.37 [CI 95% 9.40–19.00]; *COVID-19 n* = 337 ROR 1.81 [CI 95% 1.57–2.09]. Table 7, Table 8, Table 9 and Table 10 display the most frequently reported and statistically significant AEs associated with biosimilars in comparison to their originators, categorized by the number of AEs. Within these drug–event pairs, we identified serious events as outlined below, based on the Important Medical Event list [35]. Adalimumab biosimilars vs. *Humira*^®^: *psoriatic arthropathy n* = 125 events, ROR 1.88 [CI 95% 1.57–2.25]; *Crohn’s disease n* = 106, ROR 1.733 [CI 95% 1.09–1.61]; *lower respiratory tract infection n* = 54, ROR 2.11 [CI 95% 1.60–2.79]. Etanercept biosimilars vs. *Enbrel*^®^: *pemphigus n* = 372 events, ROR 1.11 [CI 95% 1.00–1.23]; *psoriatic arthropathy n* = 275, ROR 1.87 [CI 95% 1.66–2.11]; *liver injury n =* 202, ROR 3.25 [CI 95% 2.81–3.76]. Infliximab biosimilars vs. *Remicade*^®^: *haematochezia n =* 698 events, ROR 1.42 [CI 95% 1.31–1.54]; *uveitis n =* 446, ROR 2.15 [CI 95% 1.93–2.39]; *intestinal obstruction n =* 295, ROR 2.12 [CI 95% 1.85–2.42]. Rituximab biosimilars vs. *MabThera*^®^: *rheumatoid arthritis n =* 118 events, ROR 4.08 [CI 95% 2.74–6.08]; *immune thrombocytopenia n =* 66, ROR 5.12 [CI 95% 2.46–10.65]; *systemic lupus erythematosus n =* 46, ROR 4.76 [CI 95% 1.85–12.21]. Moreover, we examined the drug–event pairs with a higher and statistically significant ROR, outlined below. Adalimumab biosimilars vs. *Humira*^®^: *therapeutic response changed n =* 32 events, ROR 115.16 [CI 95% 30.56–433.94]; *administration site pain n =* 15, ROR 53.92 [CI 95% 12.20–238.35]; *rush pustular n =* 13, ROR 46.73 [CI 95% 10.18–214.45]. Etanercept biosimilars vs. *Enbrel*^®^: *smear cervix abnormal n =* 12 events, ROR 44.81 [CI 95% 3.58–560.22]; *bowel movement irregularity n =* 19, ROR 35.48 [CI 95% 8.49–148.23]; *vitamin B12 decreased n =* 9, ROR 33.61 [CI 95% 2.46–458.56]. Infliximab biosimilars vs. *Remicade*^®^: *haemoptysis n =* 26 events, ROR 19.38 [CI 95% 1.86–202.08]; *ecchymosis n =* 20, ROR 14.91 [CI 95% 1.35–164.04]; *nasal dryness n =* 17 ROR 12.67 [CI 95% 1.11–144.70]. Rituximab biosimilars vs. *MabThera*^®^: *blood pressure fluctuation n = 210* events, ROR 32.85 [CI 95% 10.76–100.23]; *blood pressure systolic increased n =* 93, ROR 28.93 [CI 95% 3.32–251.74]; *heart rate irregular n =* 78, ROR 1.81 [CI 95% 2.73–214.96].

## 4. Discussion

Biosimilars necessitate the submission to the EMA of a Risk Management Plan, inclusive of an additional safety study, for approval to be obtained. Furthermore, their safety profile is monitored through pharmacovigilance activities once they are released onto the market, akin to other medicines [4,8].

This comparative analysis of post-marketing pharmacovigilance data between biosimilars and their corresponding reference biologics, using the EudraVigilance database, has provided valuable insights into the safety profiles of these treatments in rheumatology.

We obtained and analyzed a substantial volume of reports, with 75,327 total reports, comprising 566,249 drug–event pairs. The distribution between the original products and biosimilars was 58.8% and 41.2%, respectively. During the study period, the number of reports on RPs increased in 2022 compared to the previous year, then decreased in 2023. Meanwhile, the number of reports on biosimilars has noticeably risen over the last three years, even doubling by 2023 compared to 2022. This trend is particularly noticeable for biosimilars of infliximab: *Inflectra*^®^ biosimilar reports tripled in 2023, while *Remicade*^®^ reports slightly declined (Figure 1 and Figure 2).

This distribution suggests a significant uptake of biosimilars in clinical practice, reflecting their growing acceptance as cost-effective alternatives to originator biologics and it may also reflect the response to incentive programs instituted by individual member states, health authorities, and payers, over the last few years [35,36]. The EU has been a global leader in biosimilar approval and adoption, with significant cost-saving initiatives implemented across member states [37,38]. Moreover, biosimilars being newer products may be subject to increased scrutiny by healthcare professionals and regulatory authorities, potentially leading to a higher reporting rate for AEs compared to their RPs.

The analysis reveals that more reports were generated for females for both originators and biosimilars, representing 69.4% and 56.9% of the total reports, respectively. This sex-based discrepancy could be due to the higher prevalence of autoimmune diseases in females [39], leading to greater exposure to these treatments. The age distribution of the reports shows a predominant representation of patients aged 18–64 years, followed by those aged 65–85. This trend aligns with the typical age range for chronic inflammatory and autoimmune conditions treated using these biologics [40].

Approximately 77% of the reports were submitted by healthcare professionals, indicating a high level of reliability of the data, as healthcare professionals are likely to provide more accurate and detailed reports compared to non-healthcare professionals. The differences in regard to the type of reporter may influence the observed trends in the AEs. For instance, healthcare professionals are more likely to recognize and report serious outcomes, which may have led to a higher representation of serious AEs. In contrast, non-healthcare professionals, including patients, might emphasize subjective symptoms [41], potentially influencing the disproportionality observed in regard to the events. Future studies integrating stratified analyses by reporter type could provide further insights into these differences.

The overall distribution of the AEs between biosimilars and their RPs reveals that biosimilars maintain a safety profile largely comparable to that of their originators. This is consistent with the existing literature, suggesting that biosimilars, approved through stringent regulatory pathways, demonstrate no clinically meaningful differences in terms of safety and efficacy [42].

The majority of the most frequently reported AEs for biologics were non-serious and consistent with those listed in the corresponding SPCs, which supports the EMA’s assertion that biosimilars are highly similar to their reference products in terms of safety and efficacy [4]. Our findings are in line with previous PV studies and analyses of real-world evidence, supporting the comparable safety of these medicines. For instance, Giezen et al. reported similar trends in AE reporting, particularly in the context of immunogenicity-related events and injection site events [43,44,45]. Moreover, recent meta-analyses have confirmed that biosimilars were associated with similar overall safety and immunogenicity profiles compared with their reference biologics [46,47].

However, our study identified certain AEs with statistically significant disproportionality for biosimilars compared to the originators. For instance, adalimumab biosimilars showed a higher reporting rate for injection site pain and arthralgia. These adverse events are not necessarily related to the active substance, they could result from differences in the excipients, or the injection technique, or, in the case of arthralgia, this could be a symptom of the disease for which the drug is being administered for. Therefore, this does not necessarily imply a lower safety standard, but highlights the need for continued monitoring and patient education regarding administration techniques.

The higher number of specific AEs, such as drug ineffective reports for adalimumab biosimilars, or condition aggravated for infliximab and etanercept biosimilars, and again drug effect less than expected for infliximab, warrants attention. This might reflect variability in patient responses or nocebo effects rather than true inefficacy. The nocebo effect, wherein patients experience adverse outcomes due to negative expectations, has been documented in regard to biosimilar treatments and can impact perceived efficacy [48]. Hence, improving patient and provider education about biosimilars could mitigate these perceptions and enhance the therapeutic outcomes. Effective healthcare professional–patient dialogue is key to transferring confidence to the patient, and has been shown to reduce nocebo effects in patients when switching from an originator to a biosimilar [48]. While nocebo effects may be a contributing factor, other factors, such as patient variability, prescribing practices, or disease progression, should also be considered.

Infliximab biosimilars displayed a notable increase in reports of off-label use. These findings might indicate the broader use of biosimilars in real-world settings, possibly driven by their cost effectiveness. Alternatively, it could reflect a broader acceptance of biosimilars in real-world clinical practice. However, off-label use should be evaluated with strong clinical evidence to minimize the risks.

For etanercept biosimilars, significant AEs included fatigue and alopecia. While the first one is a known ADR, alopecia is not listed in the corresponding SPC, nevertheless there are cases of alopecia areata associated with the use of etanercept reported in the literature, suggesting the need further investigations [49,50]. Differences in immunogenicity profiles could contribute to these findings. Rituximab biosimilars also exhibited higher reports of immune-related AEs, such as immune thrombocytopenia and systemic lupus erythematosus. Therefore, immunogenicity, the propensity of a drug to induce an immune response, is a crucial aspect of biosimilar evaluation, and ensuring that biosimilars undergo rigorous immunogenicity assessments during and post-approval is paramount.

Notably, our study identified some differences in the reporting of serious AEs. For example, adalimumab biosimilars showed a higher ROR for erectile dysfunction and urosepsis, and etanercept biosimilars for clear cell renal cell carcinoma and hyperadrenocorticism. All these adverse events are not listed in the corresponding SPCs; however, reports of various malignancies have been received during the post-marketing period. A possible risk in regard to the development of lymphomas, leukemia, or other hematopoietic or solid malignancies, in patients treated with a TNF antagonist cannot be excluded. Caution should be exercised when considering TNF antagonist therapy for patients with a history of malignancy or when considering continuing such treatment in patients who develop a malignancy. The other cases represented a variety of different malignancies and included rare malignancies typically associated with immunosuppression [31,32,33]. Rituximab biosimilars showed higher RORs for multiple sclerosis and mantle cell lymphoma, of which both events are unknown. However, mantle cell lymphoma is one of the indications for which rituximab is used off-label. This reported adverse event gives rise to varying interpretations. One could argue that they are merely reporting errors, with mantle cell lymphoma being the intended target disease of the rituximab, rather than an adverse event. On the other hand, it cannot be ruled out that the inclusion of the target disease among adverse events might imply disease progression due to treatment ineffectiveness. Given the substantial number of PTs and its statistically significant ROR in our dataset, the latter interpretation appears to be more plausible and warrants further investigation.

While certain findings may suggest potential differences between biosimilars and their RPs, this could also be influenced by factors such as patient perceptions, prescribing patterns, or the nocebo effect. The increased reporting of certain AEs, including drug inefficacy and injection site pain, might reflect biases in spontaneous reporting. Therefore, further research, including real-world studies and comparative effectiveness analyses, is warranted to provide a clearer assessment of biosimilar safety and efficacy.

This study’s findings are consistent with prior research indicating that biosimilars are safe alternatives to their RPs [4,51]. While biosimilars show an overall comparability to their originators, it is still essential for clinicians to monitor them for any suspected AE, particularly rare to serious ones, when treating patients with biosimilars in daily practice. Even more crucial is the reporting of any AEs encountered, in accordance with the methods and timelines established by the respective regulatory agencies. In addition, informing and educating patients about biosimilars can help resolve any concerns, reduce nocebo effects, and improve adherence. It would be beneficial for regulatory agencies and healthcare policymakers to enhance, through the use of active PV, post-marketing surveillance for biosimilars, particularly in cases of increasing off-label use or emerging safety signals; to promote educational programs for healthcare professionals and patients to address safety concerns and encourage the reporting of any suspected AEs; and to promote real-world evidence studies to further assess biosimilar safety in diverse populations.

### Strengths and Limitations

The spontaneous reporting system continues to be fundamental to pharmacovigilance, enabling the early identification of potential safety concerns and the ongoing monitoring of reported ADRs. Of particular importance is the identification of rare or long-term ADRs that may not have been detected during the limited duration and controlled conditions of premarketing clinical trials [52,53]. The spontaneous reporting system is an essential instrument for detecting previously unidentified ADRs and emerging safety concerns, as it gathers data from a wide range of healthcare providers and patients. Furthermore, post-marketing data encompass a wider patient demographic spectrum compared to the tightly regulated settings of clinical trials, incorporating individuals with co-morbidities, those taking concurrent medications, and vulnerable populations. This enables the system to reflect data on the general population and real-world clinical scenarios. However, several limitations affect this pharmacovigilance study, which uses spontaneous reporting databases. Firstly, the absence of denominator data prevents accurate calculation of incidence rates and an understanding of the true medication risks due to patient exposure. Secondly, reports often lack completeness and accuracy, potentially containing errors that undermine data reliability and validity. An important aspect to consider when interpreting such data is the potential role of the aforementioned nocebo effect. Mitigating the nocebo effect requires healthcare providers to identify patients at risk, address their concerns through a positive approach, and promote a strong patient–physician relationship to improve treatment adherence [54]. Additionally, insufficient information on patient medical histories, concomitant medications, and other factors complicates the assessment of causal relationships between suspected drugs and reported AEs. We acknowledge that the lack of adjustment for confounding factors, such as sex, age, co-morbidities, and disease types, represents a limitation in our analysis. Future studies that incorporate stratification of patient data and employ statistical methods to make adjustments in regard to these factors could provide a clearer understanding of how confounding variables influence AE reporting. The key limitation is underreporting, a phenomenon in PV that may lead to an incomplete assessment of the safety profiles of drugs. Underreporting is influenced by factors such as low awareness of PV, a lack of time, and perceived reporting burdens [55,56,57], which can skew ROR estimates and compromise data accuracy. The ROR does not establish causality, but serves to identify potential safety signals. Notably, a safety signal is essentially only a hypothesis that, together with data and arguments, justifies the need for further assessment [58]. The information reported in a signal is not conclusive, it may change substantially over time as more data accumulate.

To address these challenges, integrating the ROR with signal detection algorithms, disproportionality analyses, and additional observational or experimental studies is crucial. Future epidemiological studies, based on real-world evidence and controlled observational methodologies, will be essential to validate these findings and provide a more comprehensive understanding of biosimilar safety. These complementary approaches enhance the understanding of drug safety profiles and mitigate limitations associated with spontaneous reporting databases, ensuring more reliable pharmacovigilance outcomes.

## 5. Conclusions

In conclusion, the analysis of ADR reports underscores the safety of biosimilars as valuable alternatives to reference biologics in rheumatology.

Our findings suggest that biosimilars have safety profiles comparable to their reference products, supporting their use as cost-effective treatment options. However, continuous pharmacovigilance remains essential to monitor any potential ADRs and enhance our understanding of biosimilar safety in real-world settings. Future research should prioritize long-term safety assessments in a real-world setting to continually validate and optimize the use of biosimilars in rheumatologic care.

## Figures and Tables

**Table 1:** Biosimilars: definitions provided by international regulatory agencies.

Regulatory Authority	Definition	Reference
The European Medicines Agency (EMA)	A biosimilar is a biological medicine highly similar to another already approved biological medicine (the ‘reference medicine’). Biosimilars are approved according to the same standards of pharmaceutical quality, safety and efficacy that apply to all biological medicines.	The European Medicines Agency. Biosimilar Medicines: Overview [4]
Food and Drug Administration (FDA)	A biosimilar is a biologic medication that is highly similar to and has no clinically meaningful differences from an existing FDA-approved biologic, called a reference product.	Food and Drug Administration. Biosimilars [5]
The World Health Organization (WHO)	A biotherapeutic product which is similar in terms of quality, safety and efficacy to an already licensed reference biotherapeutic product.	The World Health Organization. Biosimilars [6]

**Table 2:** Adalimumab, etanercept, infliximab, and rituximab: reference products and biosimilar agents approved in the European Union and considered in our study.

	Reference Product	Authorization Date	Patent Expiry Date	Biosimilar Agent	Approval Date
Adalimumab	*Humira* ^®^	2003	2018	*Amgevita* ^®^	22 March 2017
				*Amsparity* ^®^	13 February 2020
				*Hulio* ^®^	17 September 2018
				*Hyrimoz* ^®^	26 July 2018
				*Hefiya* ^®^	26 July 2018
				*Idacio* ^®^	02 April 2019
				*Imraldi* ^®^	24 August 2017
Rituximab	*MabThera* ^®^	1998	2013	*Blitzima* ^®^	13 July 2017
				*Rixathon* ^®^	15 June 2017
				*Riximyo* ^®^	15 June 2017
				*Ruxience* ^®^	01 April 2020
				*Truxima* ^®^	17 February 2017
Etanercept	*Enbrel* ^®^	2000	2015	*Benepali* ^®^	14 January 2016
				*Erelzi* ^®^	23 June 2017
				*Nepexto* ^®^	20 May 2020
Infliximab	*Remicade* ^®^	1999	2015	*Flixabi* ^®^	26 May 2016
				*Inflectra* ^®^	10 September 2013
				*Remsima* ^®^	10 September 2013
				*Zessly* ^®^	18 May 2018

**Table 3:** Demographic characteristics and reporter type for ICSRs on rituximabs.

Characteristics		Rituximab					
		Originator Biologic *MabThera*^®^ (*n* = 2451)	Biosimilar				
			*Blitzima*^®^ (*n* = 9)	*Rixathon*^®^ (*n* = 736)	*Riximyo*^®^ (*n* = 78)	*Ruxience*^®^ (*n* = 2329)	*Truxima*^®^ (*n* = 1019)
Sex	Female	1270 (51.8)	2 (22.2)	419 (56.9)	59 (75.6)	1578 (67.8)	276 (27.1)
	Male	1135 (46.3)	4 (44.4)	299 (40.6)	18 (23.1)	736 (31.6)	217 (21.3)
	Unknown	46 (1.9)	3 (33.3)	18 (2.4)	1 (1.3)	15 (0.6)	526 (51.6)
Age (years)	0–17	75 (3.1)	1 (11.1)	27 (3.7)	2 (2.6)	10 (0.4)	22 (2.1)
	18–64	1114 (45.4)	3 (33.3)	369 (50.1)	36 (46.1)	1272 (54.6)	329 (32.3)
	65–85	791 (32.3)	2 (22.2)	246 (33.4)	24 (30.8)	961 (41.3)	165 (16.2)
	>85	32 (1.3)	0 (0.0)	16 (2.3)	2 (2.6)	52 (2.2)	10 (1.0)
	Unknown	439 (17.9)	3 (33.3)	78 (10.6)	14 (17.9)	34 (1.5)	493 (48.4)
Reporter type	Healthcare Professional	2248 (91.7)	9 (100)	709 (96.3)	75 (96.2)	2235 (96.0)	956 (93.8)
	Non-healthcare Professional	203 (8.3)	0 (0.0)	27 (3.7)	3 (3.8)	94 (4.0)	63 (6.2)

**Table 4:** Demographic characteristics and reporter type for ICSRs on etanercepts.

Characteristics		Etanercept			
		Originator Biologic *Enbrel*^®^ (*n* = 14,588)	Biosimilar		
			*Benepali*^®^ (*n* = 1327)	*Erelzi*^®^ (*n* = 2473)	*Nepexto*^®^ (*n =* 69)
Sex	Female	11,623 (79.7)	901 (67.9)	2003 (81.0)	55 (79.7)
	Male	2675 (18.3)	392 (29.5)	426 (17.2)	12 (17.4)
	Unknown	290 (2.0)	34 (2.6)	44 (1.8)	2 (2.9)
Age (years)	0–17	262 (1.8)	13 (0.9)	44 (1.8)	3 (4.4)
	18–64	6744 (46.2)	662 (49.9)	1272 (51.4)	29 (42.0)
	65–85	3816 (26.2)	305 (23.0)	387 (15.6)	21 (30.4)
	>85	168 (1.1)	7 (0.5)	16 (0.6)	1 (1.4)
	Unknown	3598 (24.7)	340 (25.6)	754 (30.5)	15 (21.7)
Reporter type	Healthcare Professional	7412 (50.8)	1010 (76.1)	2203 (89.1)	57 (82.6)
	Non-healthcare Professional	7176 (49.2)	317 (23.9)	270 (10.9)	12 (17.4)

**Table 5:** Demographic characteristics and reporter type for ICSRs on adalimumabs.

Characteristics		Adalimumab				
		Originator Biologic *Humira*^®^ (*n* = 14,002)	Biosimilar			
			*Amgevita*^®^ (*n* = 1835)	*Amsparity*^®^ (*n* = 23)	*Hefiya*^®^ (*n* = 10)	*Hukyndra*^®^ (*n* = 28)
Sex	Female	9689 (69.2)	1106 (60.3)	16 (69.6)	8 (80.0)	22 (78.6)
	Male	3845 (27.5)	700 (38.1)	6 (26.1)	2 (20.0)	6 (21.4)
	Unknown	468 (3.3)	29 (1.6)	1 (4.3)	0 (0.0)	0 (0.0)
Age (years)	0–17	435 (3.1)	48 (2.6)	0 (0.0)	0 (0.)	0 (0.0)
	18–64	6574 (47.0)	958 (52.2)	14 (60.9)	4 (40.0)	16 (57.1)
	65–85	1812 (12.9)	226 (12.3)	0 (0.0)	1 (10.)	7 (30.4)
	>85	58 (0.4)	7 (0.4)	0 (0.0)	0 (0.0)	0 (0.0)
	Unknown	5123 (36.6)	596 (32.5)	9 (39.1)	5 (50.0)	5 (21.7)
Reporter type	Healthcare Professional	9328 (66.6)	1205 (65.7)	18 (78.3)	5 (50.0)	22 (78.6)
	Non-healthcare Professional	4674 (33.4)	630 (34.3)	5 (21.7)	5 (50.0)	6 (21.4)
**Characteristics**		**Adalimumab**				
		**Biosimilar**				
		***Hulio*^®^ (*n* = 304)**	***Hyrimoz*^®^ (*n* = 2200)**	***Idacio*^®^ (*n* = 634)**	***Imraldi*^®^ (*n* = 1424)**	***Yuflyma*^®^ (*n* = 498)**
Sex	Female	183 (60.2)	1258 (57.2)	402 (63.4)	881 (61.9)	322 (64.7)
	Male	114 (37.5)	835 (38.0)	219 (34.5)	517 (36.3)	164 (32.9)
	Unknown	7 (2.3)	107 (4.9)	13 (2.1)	26 (1.8)	12 (2.4)
Age (years)	0–17	6 (2.0)	45 (2.0)	9 (1.4)	17 (1.2)	7 (1.4)
	18–64	179 (58.9)	1405 (63.9)	447 (70.5)	1002 (70.4)	345 (69.3)
	65–85	49 (16.1)	268 (12.2)	94 (14.8)	187 (13.1)	57 (11.4)
	>85	0 (0.0)	8 (0.4)	0 (0.0)	5 (0.4)	2 (0.4)
	Unknown	70 (23.0)	472 (21.5)	84 (13.2)	213 (15.0)	87 (17.5)
Reporter type	Healthcare Professional	234 (77.0)	1869 (85.0)	490 (77.3)	1188 (83.4)	334 (67.1)
	Non-healthcare Professional	70 (23.0)	331 (15.0)	144 (22.7)	236 (16.6)	164 (32.9)

**Table 6:** Demographic characteristics and reporter type for ICSRs on infliximabs.

Characteristics		Infliximab				
		Originator Biologic *Remicade*^®^ (*n* = 13,248)	Biosimilar			
			*Flixabi*^®^ (*n* = 592)	*Inflectra*^®^ (*n* = 12,462)	*Remsima*^®^ (*n* = 2563)	*Zessly*^®^ (*n* = 425)
Sex	Female	8166 (61.6)	306 (51.7)	6453 (51.8)	1234 (48.1)	188 (44.2)
	Male	4759 (35.9)	277 (46.8)	5736 (46.0)	816 (31.8)	221 (52.0)
	Unknown	323 (2.4)	9 (1.5)	273 (2.2)	513 (20.0)	16 (3.8)
Age (years)	0–17	335 (2.5)	45 (7.6)	191 (1.5)	71 (2.8)	17 (4.0)
	18–64	8323 (62.8)	469 (79.2)	9761 (78.3)	1368 (53.4)	308 (72.5)
	65–85	1744 (13.2)	46 (7.8)	2007 (16.1)	212 (8.3)	44 (10.4)
	>85	68 (0.5)	1 (0.2)	69 (0.6)	5 (0.2)	1 (0.2)
	Unknown	2778 (21.0)	31 (5.2)	434 (3.5)	907 (35.4)	55 (12.9)
Reporter type	Healthcare Professional	11,913 (89.9)	416 (70.3)	11,978 (96.1)	1892 (73.8)	405 (95.3)
	Non-healthcare Professional	1335 (10.1)	176 (29.7)	484 (3.9)	671 (26.2)	20 (4.7)

**Table 7:** The most frequent and statistically significant AEs related to adalimumab biosimilars compared to *Humira*^®^ reported in EV.

Adalimumab					
AE	N	N*	ROR	CI 95%_Lower	CI 95%_Upper
Drug ineffective	838	5067	1.20	1.12	1.28
Injection site pain	450	342	9.70	8.79	10.70
Arthralgia	440	2095	1.52	1.39	1.67
Psoriasis	396	587	4.94	4.46	5.47
Pruritus	231	344	4.88	4.26	5.58
Headache	223	989	1.63	1.43	1.86
Arthritis	218	530	2.98	2.60	3.42
Condition aggravated	200	1224	1.18	1.02	1.35
Loss of therapeutic response	146	35	30.23	24.30	37.61
Product substitution issue	141	8	127.72	84.75	192.50
COVID-19	138	394	2.53	2.13	3.00
Urticaria	137	233	4.25	3.57	5.07
Erythema	133	323	2.97	2.49	3.55
Psoriatic arthropathy	125	479	1.88	1.57	2.25
Skin exfoliation	113	52	15.71	12.58	19.62
Pyrexia	111	433	1.85	1.53	2.24
Crohn’s disease	106	575	1.33	1.09	1.61
Dizziness	105	334	2.27	1.86	2.76
Abdominal pain	105	476	1.59	1.31	1.93
Malaise	104	611	1.22	1.01	1.49
Asthenia	99	374	1.91	1.56	2.33
Dyspnea	99	453	1.57	1.29	1.92
Back pain	97	347	2.01	1.64	2.47
Therapy non-responder	94	126	5.38	4.33	6.70
Injection site erythema	94	177	3.83	3.10	4.74
Paraesthesia	84	143	4.24	3.37	5.32
Myalgia	80	157	3.67	2.91	4.63
Cough	79	182	3.13	2.48	3.94
Vomiting	61	316	1.39	1.07	1.80
Anxiety	58	216	1.93	1.48	2.52

AE adverse event, N number of suspected AEs in regard to biosimilars, N* number of suspected AEs in regard to *MabThera*^®^, ROR reporting odds ratio, CI 95%_lower lower limit of the 95% confidence interval, CI 95%_upper upper limit of the 95% confidence interval.

**Table 8:** The most frequent and statistically significant AEs related to etanercept biosimilars compared to Enbrel^®^ reported in EV.

Etanercept					
AE	N	N*	ROR	CI 95%_Lower	CI 95%_Upper
Fatigue	685	2190	1.17	1.09	1.26
Alopecia	615	1720	1.34	1.24	1.45
Arthropathy	551	1680	1.23	1.13	1.34
Abdominal discomfort	541	1662	1.22	1.12	1.33
Glossodynia	471	1227	1.44	1.31	1.58
Condition aggravated	429	1297	1.24	1.12	1.36
Injection site pain	374	283	4.98	4.47	5.55
Pemphigus	372	1250	1.11	1.00	1.23
Hand deformity	370	1114	1.24	1.12	1.38
Product use issue	353	845	1.57	1.41	1.74
Discomfort	350	830	1.58	1.42	1.76
Infusion-related reaction	312	865	1.35	1.21	1.51
General physical health deterioration	295	434	2.55	2.27	2.87
Musculoskeletal stiffness	289	803	1.35	1.20	1.51
Psoriatic arthropathy	275	551	1.87	1.66	2.11
Maternal exposure during pregnancy	273	868	1.18	1.04	1.33
Anti-cyclic citrullinated peptide antibody positive	270	685	1.48	1.31	1.67
C-reactive protein increased	229	445	1.93	1.69	2.20
Blister	226	734	1.15	1.01	1.31
Blood cholesterol increased	211	379	2.09	1.81	2.40
Arthritis	209	469	1.67	1.45	1.92
Confusional state	207	618	1.25	1.09	1.44
Liver injury	202	233	3.25	2.81	3.76
Duodenal ulcer perforation	175	424	1.54	1.32	1.80
Helicobacter infection	159	434	1.37	1.17	1.61
Product use in unapproved indication	155	141	4.12	3.47	4.89
Psoriasis	153	222	2.58	2.18	3.05
Exposure during pregnancy	152	234	2.43	2.06	2.87
Dizziness	152	256	2.22	1.88	2.63
Intentional product use issue	150	411	1.36	1.16	1.61

AE adverse event, N number of suspected AEs in regard to biosimilars, N* number of suspected AEs in regard to *Enbrel*^®^, ROR reporting odds ratio, CI 95%_lower lower limit of the 95% confidence interval, CI 95%_upper upper limit of the 95% confidence interval.

**Table 9:** The most frequent and statistically significant AEs related to infliximab biosimilars compared to *Remicade*^®^ reported in EV.

Infliximab					
AE	N	N*	ROR	CI 95%_Lower	CI 95%_Upper
Off-label use	10,028	5703	1.33	1.31	1.36
Condition aggravated	7281	3110	1.78	1.74	1.83
Intentional product use issue	6020	2428	1.88	1.84	1.93
Inappropriate schedule of product administration	4449	1322	2.56	2.48	2.64
Arthralgia	3439	1942	1.33	1.28	1.37
COVID-19	2173	559	2.93	2.80	3.06
Weight increased	2086	1035	1.51	1.44	1.58
Malaise	2006	771	1.95	1.86	2.04
Weight decreased	1913	808	1.77	1.69	1.86
Blood pressure increased	1646	515	2.40	2.28	2.53
Headache	1628	1003	1.21	1.15	1.27
Nausea	1544	812	1.42	1.35	1.50
Cough	1292	336	2.88	2.71	3.06
Therapeutic response shortened	1248	152	6.16	5.76	6.60
Nasopharyngitis	1230	392	2.35	2.21	2.50
Blood pressure fluctuation	1188	396	2.25	2.11	2.39
Heart rate decreased	1184	345	2.57	2.41	2.74
Pruritus	1152	454	1.90	1.78	2.02
Incorrect dose administered	1133	346	2.45	2.30	2.61
Dyspnoea	1099	584	1.41	1.32	1.50
Pyrexia	1089	468	1.74	1.63	1.85
Abdominal pain	951	631	1.12	1.05	1.20
Erythema	937	371	1.89	1.76	2.02
Dizziness	930	397	1.75	1.63	1.88
Drug level decreased	828	98	6.33	5.79	6.91
Oropharyngeal pain	826	203	3.04	2.82	3.29
Drug level below therapeutic	809	194	3.12	2.88	3.38
Vomiting	804	374	1.61	1.49	1.73
Asthenia	740	374	1.48	1.37	1.60
Back pain	700	266	1.97	1.81	2.13

AE adverse event, N number of suspected AEs in regard to biosimilars, N* number of suspected AEs in regard to *Remicade*^®^, ROR reporting odds ratio, CI 95%_lower lower limit of the 95% confidence interval, CI 95%_upper upper limit of the 95% confidence interval.

**Table 10:** The most frequent and statistically significant AEs related to rituximab biosimilars compared to *MabThera*^®^ reported in EV.

Rituximab					
AE	N	N*	ROR	CI 95%_Lower	CI 95%_Upper
Off-label use	2064	382	1.75	1.67	1.84
Intentional product use issue	590	31	6.04	5.23	6.98
Condition aggravated	340	8	13.37	9.40	19.00
COVID-19	337	58	1.81	1.57	2.09
Pruritus	320	59	1.69	1.46	1.95
Throat irritation	290	14	6.49	5.03	8.38
Nausea	261	59	1.37	1.18	1.61
Headache	259	23	3.52	2.86	4.33
Malaise	258	11	7.34	5.44	9.92
Fatigue	245	32	2.39	1.98	2.88
Inappropriate schedule of product administration	243	2	38.07	12.59	115.07
Blood pressure fluctuation	210	2	32.85	10.76	100.23
Hypertension	206	43	1.49	1.24	1.79
Pain	199	15	4.14	3.16	5.42
Cough	199	25	2.48	1.99	3.08
Blood pressure increased	196	42	1.45	1.20	1.75
Product use issue	194	2	30.32	9.88	93.03
Erythema	178	30	1.84	1.49	2.28
Arthralgia	172	20	2.68	2.09	3.43
Dizziness	144	24	1.86	1.46	2.38
Rheumatoid arthritis	118	9	4.08	2.74	6.08
Urticaria	114	23	1.54	1.17	2.01
Weight decreased	103	10	3.20	2.17	4.73
Blood pressure systolic increased	93	1	28.93	3.32	251.74
Throat tightness	91	7	4.04	2.48	6.57
Nasopharyngitis	90	5	5.59	3.07	10.18
Pain in extremity	90	7	3.99	2.45	6.50
Ear pruritus	88	2	13.68	4.16	44.94
Illness	86	3	8.91	3.75	21.16
Weight increased	84	7	3.73	2.27	6.11

AE adverse event, N number of suspected AEs in regard to biosimilars, N* number of suspected AEs in regard to *MabThera*^®^, ROR reporting odds ratio, CI 95%_lower lower limit of the 95% confidence interval, CI 95%_upper upper limit of the 95% confidence interval.

## Data Availability

The data that support the findings of this study are available from the corresponding author upon reasonable request.

## Abbreviations

The following abbreviations are used in this manuscript:

ADR	Adverse drug reaction
AE	Adverse event
EMA	European Medicines Agency
EU	European Union
EV	EudraVigilance
FDA	United States Food and Drug Administration
mAb	Monoclonal antibody
MedDRA	Medical Dictionary for Regulatory Activities
PTs	Preferred terms
RPs	Reference products
ROR	Reporting odds ratio
SPCs	Summary of Product Characteristics
